# Molecular Mechanism of Action of Neonicotinoid Insecticides

**DOI:** 10.3390/ijms24065484

**Published:** 2023-03-13

**Authors:** Steeve H. Thany

**Affiliations:** Department of Biology and Biochemistry, Université d’Orléans, LBLGC USC-INRAE 1328, 1 rue de Chartres, 45067 Orléans, France; steeve.thany@univ-orleans.fr; Tel.: +33-2-38-41-70-58

Since neonicotinoid insecticides were first introduced several years ago, most of them have been banned by the European Union due to their potentially adverse effects on humans and useful insects. This Special Issue is a collection of six articles that explore the molecular mode of action of neonicotinoid insecticides on insects and mammals. The contributions cover a diversity of questions, the authors varying in their approaches. Neonicotinoid insecticides are used worldwide and have been found to be effective against a broad range of insect pests. They represent a group of chemicals that includes compounds such as imidacloprid, clothianidin, thiacloprid, and acetamiprid. Several studies have revealed that they are toxic to useful insects such as honeybees [1,2,3]. In their review, Chen et al. describe the chronic effects that imidacloprid has on honeybee worker development. In particular, they reported that sublethal imidacloprid treatment during the larval stage causes changes to gene expression in larvae, pupae, and adults. Interestingly, they provided arguments on how sublethal doses of imidacloprid impair bees’ olfactory-associative learning ability, reducing their activity and social interaction. Finally, they discussed the molecular impact of imidacloprid on other pollinators. Their results improve our understanding of how the mode of action of sublethal concentrations of imidacloprid could affect bee social interactions. In another review, Malhotra and colleagues explored the effects of neonicotinoids on non-target aquatic invertebrates. They found that neonicotinoids are significatively toxic to an extensive variety of aquatic invertebrates and demonstrated that there is a lack of knowledge surrounding the toxicity of neonicotinoids to vertebrate aquatic species. Indeed, very few studies refer to the molecular mechanisms of neonicotinoid action [4,5], which has yet to be characterized in aquatic invertebrates. Aquatic invertebrates tend to inhabit freshwater ecosystems in proximity to agricultural regions and are unintentionally exposed to neonicotinoids.

This issue also explores the agonist effects of neonicotinoids on insect nicotinic acetylcholine receptors (nAChRs) subtypes. nAChRs are of particular interest because they are involved in rapid neurotransmission in many species, from arthropods to mammals [6,7]. Most nAChRs are heteropentamers consisting of α subunits, with a pair of adjacent cysteines in loop C of the extracellular N-terminal region, or ligand binding domain, and non-α subunits (β subunits), without this motif. Homopentamers composed of α subunits are known as homomeric α receptors [8,9]. Several studies have proven that insects and mammals express both heteromeric and homomeric neuronal nAChRs [7]. They participate in the mode of action of neonicotinoids [10,11], and studying their molecular mechanisms may contribute to understanding the selectivity of neonicotinoids for insect and mammalian neuronal nAChRs. Unfortunately, the mode of action of neonicotinoids on insect nAChR subtypes is somewhat difficult to study. Cartereau et al. used the cockroach homomeric nAChR subtype, Pameα7, to demonstrate that imidacloprid was not able to activate the Pameα7 subtype, whereas thiacloprid was able to induce a lower current amplitude than nicotine. Imidacloprid and thiacloprid showed similar trends, due to the pyridine ring of the two ligands forming π-π interactions with Trp189 of cockroach Pameα7 nAChRs. In concordance with experimental data, the stability of the two ligands inside the binding pocket appears to be realized through CH…π and π…π interactions. This involves positively polarized CH groups of the five-membered saturated ring of the ligands, and aromatic amino acid residues of the cockroach Pameα7 nAChR, such as Trp189 and Tyr231. The discrepancy between the electrophysiological and the docking model appears to be linked to other residues in the binding pocket. The aim of using specific insect nAChR subtypes is to find the best target for new active compounds. In their study, Qu et al., suggested that co-delivery of neonicotionoids and dsRNA targeting genes can be used as a strategy to control pest insects. In particular, they studied the sublethal effects of thiamethoxam on gene expression through RNA sequencing in the melon aphid *Aphis gossypii*. Their goal was to demonstrate that when synapsin is knocked down, aphid mortality can be induced. Indeed, synapsin and the endocytic scaffolding complex regulate synaptic vesicle clustering. In their results, they found that 1106 genes were upregulated and 699 genes downregulated in aphids exposed to the LC50 concentration of TMX. These genes are mainly involved in the metabolism and cellular processes, which is the case for synapsin. Using this method, we will be able to study gene regulation during the sublethal application of neonicotinoid insecticides.

The mode of action of neonicotinoids on mammals appears to be more complex. In their review, Costas-Ferreira and colleagues proposed that the presence of neonicotinoids in the environment could increase the risk of exposure and toxicity. They discussed previous studies exploring the effect of neonicotinoids on neuronal development, cell migration, and neuroinflammation. Indeed, neonicotinoids have chloropyridylmethyl, chlorothiazolylmethyl, or tetrahydrofuranylmethyl substituents, which can induce several metabolites [12,13,14]. Desnitroimidacloprid, a bioactivation product of imidacloprid, binds to the same site as imidacloprid on rat α4β2 receptors with high affinity [15], and the cytosolic aldehyde oxidase is known to enhance imidacloprid potency at α4β2 receptors [16], probably due to a partial conversion of the nitroguanidine to a more potent metabolite [17,18]. It seems that these metabolites do not have the same binding affinity and mode of action on mammalian nAChRs. However, several lines of evidence have demonstrated that neonicotinoid insecticides can modulate cholinergic functions through neuronal nAChRs. Major studies on the influence of neonicotinoid insecticides on cholinergic functions have been conducted using homomeric α7 and heteromeric α4β2 receptors, as they are the most abundant in the nervous system. Several studies have demonstrated that neonicotinoid insecticides induce small direct activation responses from mammalian neuronal nAChRs with low affinity or low efficacy. For example, clothianidin is a low-affinity agonist of the human α4β2 and is ineffective in inhibiting responses elicited by ACh, whereas responses to low concentrations of ACh are potentiated by clothianidin [11]. It was suggested that clothianidin binds to at least one ACh-binding site on the α4β2 receptors [11]. With the same receptor, imidacloprid appears to be a low-efficacy but high-affinity agonist [11]. In other studies, imidacloprid was unable to activate the chicken α4β2 receptors, but co-application with ACh potentiated the response to 1 µM ACh [19], which is in accordance with the finding that [^3^H]imidacloprid has a very low binding affinity for rat α4β2 [20]. In other studies, Ihara et al. found that neonicotinoids with a nitroimine or nitromethylene group, such imidacloprid and nitenpyram, are inactive as agonists on the α4β2 receptors, although they can influence ACh-evoked responses [21]. At a low concentration imidacloprid potentiated ACh-induced responses whereas nitenpyram suppressed the responses [21]. The effects of imidacloprid can be enhanced or diminished following specific mutations in the C and D loops of the chicken α4β2 subtype [22]. Thus, of the six loops (A-F) composing the ACh binding site, the C and D loops are most likely to contribute strongly to the interactions with imidacloprid, as well as the subunit stoichiometry. Evidence for the modulatory actions of neonicotinoids on mammalian nAChRs has also been observed on the homomeric α7 receptors. Imidacloprid and nitenpyram induce rapid and desensitized currents on chicken α7 receptors suggesting that they are partial agonists of these receptors [21]. Clothianidin and acetamiprid increase ACh-evoked currents, whereas at low concentrations, thiamethoxam decreases the ACh-induced currents on rat α7 receptors [23]. The concentration-response curve to ACh was right-shifted by pretreatment with thiamethoxam. The effects of different pretreatment concentrations of thiamethoxam reveal only a partial inhibition of the ACh-evoked currents, whereas coapplication completely blocked nicotine-induced currents [23]. These results concerning heteromeric α4β2 and homomeric α7 receptors lead us to propose that neonicotinoid insecticides have a modulatory activity that can be compared to an agonist effect. In their manuscript, Park et al. had substantial information on the mode of action of neonicotinoids on other nAChR subtypes. They suggested that the partial agonist activity of clothianidin and acetamiprid on α3β4 nAChRs could have consequences on epinephrine secretion and in vivo blood pressure. In their results, they found that at higher concentrations, clothianidin and acetamiprid stimulated epinephrine secretion, which was due to the activation of α3β4 in the adrenal medulla glands.

To conclude, this issue demonstrates that understanding the pharmacological mechanism of action of neonicotinoid insecticides is of particular interest in the context of designing new insecticides. The discovery of these diverse actions of neonicotinoid insecticides on insect and mammalian neuronal nAChR subtypes not only provides evidence of their complex action in terms of their toxic effects, but also provides novel insights into nAChR-ligand interactions.
